# Human movement in simulated hypogravity—Bridging the gap between space research and terrestrial rehabilitation

**DOI:** 10.3389/fneur.2023.1062349

**Published:** 2023-02-06

**Authors:** Enrico De Martino, David A. Green, Daniel Ciampi de Andrade, Tobias Weber, Nolan Herssens

**Affiliations:** ^1^Department of Health Science and Technology, Center for Neuroplasticity and Pain, Faculty of Medicine, Aalborg University, Aalborg, Denmark; ^2^Space Medicine Team, European Astronaut Centre, Cologne, Germany; ^3^KBR GmbH, Cologne, Germany; ^4^Centre of Human and Applied Physiological Sciences, King's College London, London, United Kingdom

**Keywords:** hypogravity, body weight support, neurorehabilitation, orthopedic rehabilitation, spaceflight, exercise, reconditioning

## Abstract

Human movement is optimized to Earth's gravity and based on highly complex interactions between sensory and neuro-muscular systems. Yet, humans are able to adapt—at least partially—to extreme environments upon and beyond Earth's surface. With upcoming Lunar Gateway and Artemis missions, it is crucial to increase our understanding of the impact of hypogravity—i.e., reduced vertical loading—on physiological and sensory-motor performances to improve countermeasure programs, and define crewmember's readiness to perform mission critical tasks. Several methodologies designed to reduce vertical loading are used to simulate hypogravity on Earth, including body weight support (BWS) devices. Countering gravity and offloading the human body is also used in various rehabilitation scenarios to improve motor recovery in neurological and orthopedic impairments. Thus, BWS-devices have the potential of advancing theory and practice of both space exploration and terrestrial rehabilitation by improving our understanding of physiological and sensory-motor adaptations to reduced vertical loading and sensory input. However, lack of standardization of BWS-related research protocols and reporting hinders the exchange of key findings and new advancements in both areas. The aim of this introduction paper is to review the role of BWS in understanding human movement in simulated hypogravity and the use of BWS in terrestrial rehabilitation, and to identify relevant research areas contributing to the optimization of human spaceflight and terrestrial rehabilitation. One of the main aims of this research topic is to facilitate standardization of hypogravity-related research protocols and outcome reporting, aimed at optimizing knowledge transfer between space research and BWS-related rehabilitation sciences.

## 1. Introduction

Human bi-pedal locomotion, upright postural control and movement have adapted for performance in Earth's gravity (1g) based on complex interactions between the sensory and neuro-muscular systems (1, 2). Adaptation is extremely dynamic as humans are able to (at least partially) adapt to novel sensory and functional conditions due to impairment, such as with transtibial amputation (3) or stroke (4), in addition to environmental changes, such as load carrying (5) or walking on uneven surfaces (6). Human movement has also been shown to adapt to microgravity (μg), although such changes may present issues post-flight as modulation of sensori-motor (7, 8) and upright postural control (9) can persist for a number of days following the return to Earth's gravity (10, 11).

Whilst images of Apollo astronauts “hopping” across the Lunar surface are in the public consciousness, little is known about the transition from 1 g or μg to *hypogravity* (e.g., Moon: 0.16 g, Mars: 0.38 g) (11, 12). This is critical as the upcoming Lunar Gateway (a crewed space station orbiting the Moon) and Artemis missions (missions designed to land humans on the Moon) will mean that crewmembers will transition to Lunar gravity, potentially after prolonged exposure to μg (13). Even with limited exposure to μg (~3 days), it is reported that across the Apollo 11–17 missions, during a total of 78 h of Extra Vehicular Activities (EVAs), 23 falls, and 11 “near” falls were observed (14). The causes of Lunar instability are unknown, although novel and dynamic factors within the hypogravity environment presumably include the Lunar surface characteristics, the EVA suit (including the Portable Life Support System; PLSS) and the challenge of controlling the Center of Mass (CoM) with respect to the base of support (Center of Pressure) (15). Such factors are compounded by vestibular adaptations (16) leading to impairment of gross (i.e., postural control and locomotion) motor control, in addition to motion sickness and spatial disorientation (14). As a result, the risk of injury and/or fatality has been considered to be high on the Lunar surface (14, 17).

The Artemis missions may involve sustained (and potentially repeated) exposure to μg that may extend to many weeks/months (13). As a result, physiological adaptations associated with sustained exposure to μg may be induced, including musculoskeletal (18, 19) and cardiovascular deconditioning (20). Furthermore, changes in sensory-motor control (14) may reflect recently identified neuroplasticity (21, 22) including cortical reorganization (23). Thus, increasing the understanding of the impact of hypogravity (i.e., < 1 g; >μg) on physiological and sensory-motor function is critical to inform development of pre-flight, in-flight, and post-flight programs to facilitate mission appropriate adaptions. An improved understanding of the control of movement in hypogravity is also key to guide the development of Lunar (and Martian) EVA suit, habitat, and general operation ergonomics and both the need for, and the nature of exercise countermeasures beyond the ISS (24) that may require the definition of “required standards” in order to ensure a crewmember's readiness to perform mission critical tasks. Whilst such exercise countermeasures are yet to be defined, hypogravity hopping appears to be a prime candidate (25).

Both in preparation for and following the Apollo program, several ground-based methodologies have been used to simulate hypogravity. The vast majority of hypogravity locomotion research has employed gravity “compensation” or “offloading” systems such as vertical body weight support (BWS) (26), tilted BWS devices (27), supine suspension (28), or lower body positive pressure (LBPP) (29) although the apparent biomechanical and physiological effects of simulated hypogravity differ—suggesting methodological-specific factors (12, 30). “Offloading” has also been used as a clinical rehabilitation tool. Body weight support has been shown to enable physically or neurologically impaired individuals to start movement rehabilitation at an earlier stage following immobilization (e.g., trauma or surgery) or improve movement in patients affected by various neuro-muscular disorders, both by reducing weight-bearing, but also by providing balance support reducing both the real risk, and the fear of falling that can limit both the willingness of a patient to move (31, 32). Thus, the appropriate use of BWS has the potential to significantly contribute to the advancement of the theory and practice of human space exploration and terrestrial rehabilitation of physically and/or neurologically impaired individuals.

Considering the above, the aims of this paper are to: (1) Review the role of BWS in understanding human movement in simulated hypogravity; (2) Review the use of BWS in terrestrial rehabilitation; (3) Identify research areas which may contribute to the optimization of human spaceflight operations and terrestrial rehabilitation. Ultimately, all three aims facilitate a standardization of hypogravity research protocols and outcome reporting to optimize knowledge transfer between studies of both space research and BWS-related rehabilitation sciences.

## 2. Use of BWS in simulated hypogravity research

**Figure 1:**
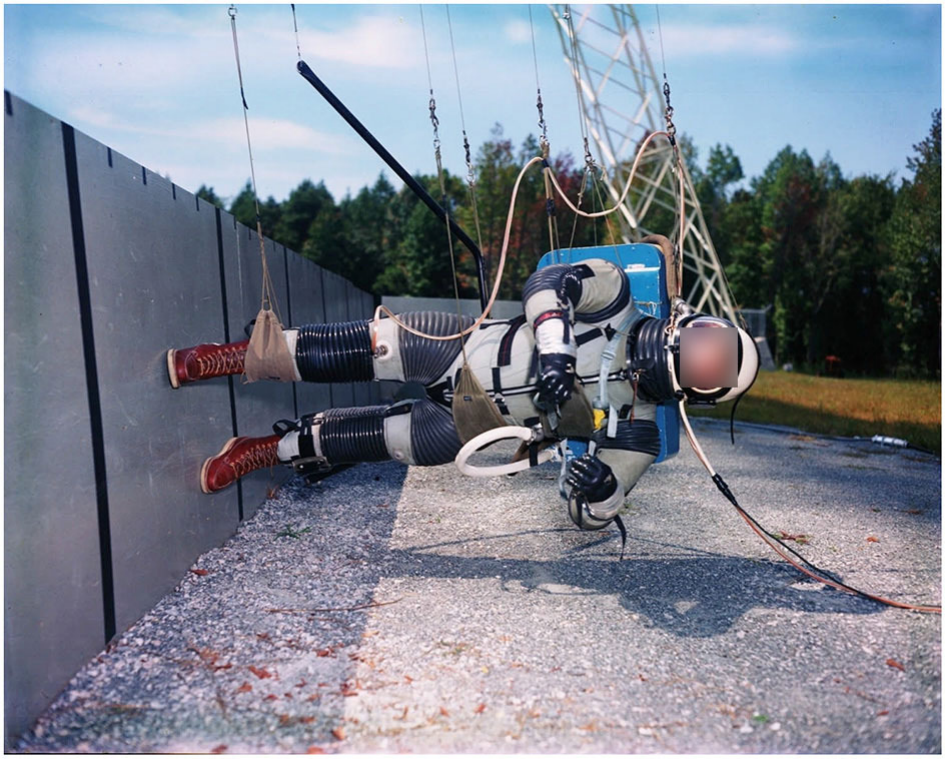
“Lunar Landing Walking Simulator” at NASA Langley Research Center (1965): Enabling researchers to study the ability to walk, run and perform other tasks required during Lunar exploration. Photo courtesy of NASA.

Simulating Lunar gravity is, in theory, fairly simple in that it requires the “unloading” of five-sixths of Earth's gravitational forces acting on the human body. Hewes and Spady (33), in preparation for the Apollo program, developed a “Lunar Landing Walking Simulator” (Figure 1) based on the assumption that: (1) if body segments are constrained to move freely in only parallel planes, subjects should be able to perform tasks in a quasi-normal manner in the direction of the planes; and (2) movement of a body or object in an inclined plane with negligible friction is only controlled by the gravitational component of the plane. The Lunar Landing Walking Simulator required individuals to be suspended *via* suspension cables whilst being inclined on their side at 9.5° from the horizontal and standing on a similarly inclined walkway (33, 34). Thus, whilst individuals were free to perform any form of locomotion along the runway (sagittal plane), lateral or rotating movements were not possible. Despite this limitation, the Lunar Landing Walking Simulator provided to be practical and invaluable to prepare the Apollo crews for their Lunar surface activities by predicting that “hopping” would be an efficient locomotory strategy on the Lunar surface, and *via* estimating the metabolic cost of locomotion in Lunar gravity to determine the oxygen requirements of the Apollo PLSS for Lunar surface EVAs (33). However, the demise of the Apollo program led to the de-commissioning of the Lunar Landing Walking Simulator, which was complex to maintain, including the outdoor walkway and a dolly system mounted to a neighboring building. Subsequently, technological advances have resulted in a variety of hypogravity locomotion simulators being developed: ranging from “simple” (e.g., vertical BWS devices) to highly complex (e.g., 3D robotic BWS systems), each with specific advantages and limitations (12, 30).

Hypogravity simulators reduce the force(s) acting on the body's CoM, affecting in particular the lower limbs, and significantly decreasing kinetic parameters (e.g., peak vertical ground reaction forces—vGRFs) which are critical for the generation of repetitive locomotor output (35, 36). Interestingly, even though peak vGRFs at simulated 0.05 g are 1/20th of those in 1 g and tend to be applied only through the forefoot rather than following the classic heel-to-toe transfer, the kinematics of the ankle, knee and hip joints remain remarkably similar, resulting in a preserved foot trajectory (shape and variability). In contrast, in the absence of contact forces during air-stepping at 100% BWS, inter-stride variability is significantly increased (12, 37). Thus, preservation of accurate lower limb kinematics and foot trajectory control appears possible in a wide range of gravity levels, including 0.05 g, as long as contact forces provide temporal signals that modulate the central pattern generators (CPGs) activity (36, 37).

**Figure 2:**
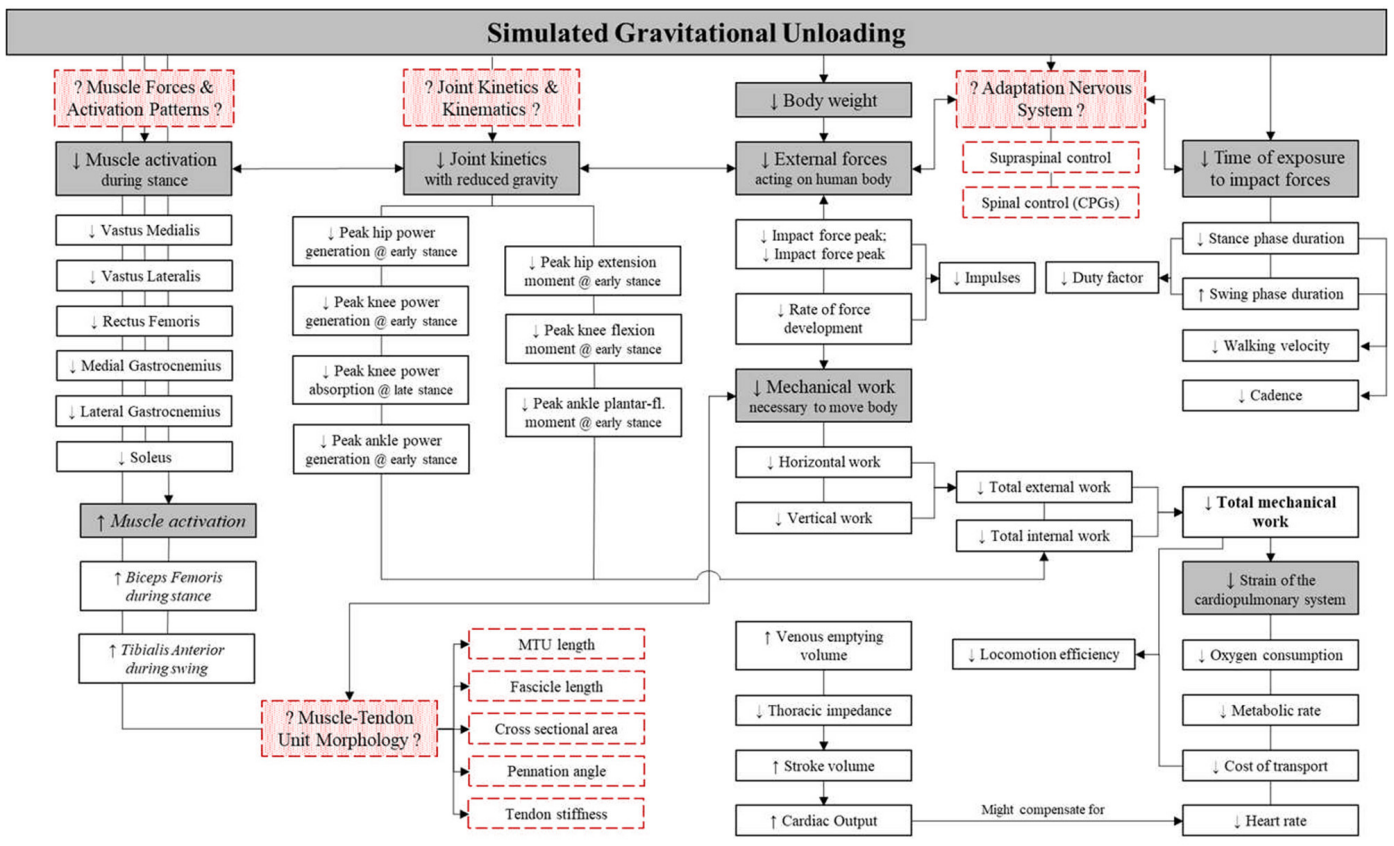
Schematic of the apparent effect of body weight support (BWS) upon lower limb muscle activation, whole body kinetics and kinematics and cardiorespiratory outcomes. Red boxes with dashed outline represent gaps in current knowledge to be filled. Gray boxes represent main physiological factors, with white boxes representing the underlying outcome parameters. Italics, trends; ↓, decrease; ↑, increase; CPG, central pattern generator; MTU, muscle-tendon unit. Figure adapted from Richter et al. (30).

However, with reduced external forces acting on the body, walking velocity, cadence and stance phase duration progressively decline and lower the rate of force development. Reductions of gravity-related mechanical load, reduce total external work (to move the body) and internal work (to move body segments) requirements, thereby reducing metabolic cost and thus cardiopulmonary demand [Figure 2; (30)]. Reductions of vGRF also reduce lower limb net joint moments and power during the stance phase (38). Reduced joint power also suggests attenuation of muscle “work” or activation, and indeed, BWS appears to result in—non-linear—decreases in knee extensor and ankle plantarflexion EMG activity during stance. Yet, knee flexor activity tended to increase during the stance phase, along with ankle dorsiflexors during the swing phase (38, 39).

However, muscle forces, joint angular velocities and joint torques, which are key to indicate internal work, have been understudied in hypogravity biomechanical research (30). Additionally, increasing the body of evidence of hypogravity research may also aid in the design of EVA suits, e.g., suit joint performance and behavior and PLSS oxygen requirements. Furthermore, outcomes related to the generation and transmission of forces across the muscle-tendon unit, such as muscle-tendon unit length, fascicle length, cross sectional area and pennation angles are critical to understand the internal kinetics of locomotion (40–42) and in particular strain, and strain rates that have been identified to be key in regulating musculoskeletal integrity (43). Such data is fundamental for modeling internal forces, to determine related de-conditioning risk of the musculoskeletal system, and to devise appropriate training and countermeasure programs, should they be needed. In addition, although modulation of activity of the primary sensory-motor areas has been reported to precede the loading and unloading of the lower limbs (44, 45), it is still unclear how reduction of external forces relates to involvement of supraspinal structures in the control of postural and lower limb musculature (46). Moreover, sensory feedback has been noted to be critical for the modulation and adaptation of CPG-generated motor output to environmental constraints, yet the role of primary afferent inputs in the control of bipedal locomotion is still only partially understood (47).

## 3. Use of BWS in terrestrial rehabilitation

The application of BWS in terrestrial rehabilitation was initially studied as an intervention to promote locomotion in spinal cord injury (SCI) patients (48). The core rationale for this approach (based on work in cordotomized cats that revealed marked locomotor improvements after a few weeks of BWS treadmill walking) was to stimulate spinal CPGs by generating cyclic locomotor patterns with reduced tonic postural contractions due to body weight support (49). Progressive reductions in BWS (starting from 60% BWS or greater) during locomotor training with incomplete SCI were employed as locomotor performance improved (50). However, whilst BWS training appears not to be superior to traditional physiotherapy and over-ground training in randomized clinical trials (51), those with the greatest impairment may particularly benefit (50, 52), presumably due to being able to perform locomotor activity without having to bear 100% body weight without risk of falling (52). More recently, BWS have been evaluated as a rehabilitation strategy to improve gait, posture and balance in patients with other neurological disorders, including stroke (53), Parkinson's disease (54), multiple sclerosis (55), and cerebral palsy (56). Whilst few well-designed, randomized clinical trials exist, BWS treadmill training is becoming increasingly popular in neurological rehabilitation in attempts to promote activity-dependent neuroplasticity. Locomotion requires the integration of descending cortical and subcortical control with CPG activity in the spinal cord along with modulatory afferent proprioceptive, mechanoreceptive and cutaneous stimuli (57).

BWS treadmill training facilitates a greater number of steps and thus task-specific stimuli within a training session. To date, there are some promising data using 30% BWS showing walking speed and endurance improvements in multiple sclerosis (55), and improvement of walking performance in Parkinson's disease (54). In contrast, BWS locomotor training (minimum 10% BWS) was not shown to be superior to progressive physiotherapist-guided home exercise in stroke patients (58) or children with cerebral palsy (56). Thus, definition of appropriate BWS-use in neurological rehabilitation remains a subject of debate (54, 59). In part this is due to a paucity of knowledge of BWS optimization (e.g., BWS methodology, BWS %, frequencies, durations, training intensities) with respect to pathological conditions (nature and severity of neurological damage) and patient-specific characteristics [e.g., age, sex, anthropometrics (including leg length)] (51, 53).

In recent years, BWS applications have been expanded to non-neurological disorders, including lower extremity injuries (60), back pain (61), and cardiac rehabilitation (29, 62). By reducing the % of body weight of such patients, locomotor training can be progressively increased to promote lower muscle strength, motor coordination, and cardiorespiratory function in respect to functionality (63). For instance, following lower limb surgery, a period of restricted weight-bearing is advised to allow surgical site healing, but attempts to ameliorate post-operative inactivity-induced results in muscular atrophy, joint stiffness, and increased thrombosis risk (64). Body weight supported walking by reducing internal joint kinetics, and muscle-tendon strain, and strain rates upon the skeletal system with minimal alteration of gait kinematics, may potentially enhance recovery following lower limb surgery or recovery of knee osteoarthritis patients, but optimal strategies remain to be determined (65). Regular 40% BWS walking has been shown to be a safe, user-friendly mode of exercise that can be used in the management of day-to-day joint symptoms associated with knee osteoarthritis (66). Furthermore, preliminary evidence suggests that BWS walking, starting from approximal 30% BWS and gradually reducing support, can promote the cardiovascular fitness, promoting autonomic regulation in patients with reduced mobility (62, 67). Finally, 40% BWS treadmill gait training promoted overground walking speed, even in healthy, older individuals (68) without increasing energy cost (69). In summary, whilst the use of BWS devices has shown potential in several rehabilitation settings, standardized randomized clinical trials are necessary to define optimal strategies (12, 70).

## 4. Bridging the gap between simulated hypogravity research and terrestrial rehabilitation

The use of BWS devices to reduce mechanical loading, thus mimicking exposure to a reduced gravitational loading—e.g., Lunar (0.16 g; 84% BWS) or Martian (0.38 g; 62% BWS) gravity—, is an invaluable tool for modeling adaptations crewmembers may experience during future surface exploration missions. However, the underlying biomechanical and neurophysiological mechanisms to these adaptations, as well as appropriate exercise countermeasures to counter and/or prevent maladaptation need to be investigated further. Importantly, the resulting findings also aid in enhancing terrestrial rehabilitation strategies in patients with various neuro-muscular and orthopedic disorders who may benefit from BWS locomotion training.

Due to a growing availability and great diversity in BWS devices, there is also a great variety of methodologies and conditions in which studies are performed. For example, the type of body weight unloading being used and related accuracy in mimicking the biomechanical and physiological effects of hypogravity, the amount of body weight unloading and corresponding simulated (hypo-)gravity level, or the mode of locomotion under investigation. In addition, the abundance of outcomes characterizing human movement complicates the comparison of results over different studies and drawing of general conclusions. Therefore, to ensure high quality and basic comparability between future studies, standardization of conditions used in BWS-related research, as well as determining a standard set of outcome measures to be used in future studies—as done for bed rest studies (71)—seems appropriate. Doing so enables greater scientific advancements, while also increasing the efficiency and added value of the scientific community's investment by ensuring a minimum set of standardized data are being reported by each study.

Thus, this Research Topic seeks to cover research areas aiding in the standardization and improvement of hypogravity-related research and training protocols, and reporting of data/outcomes. Relevant research areas include, but are not limited to:

- Improving the general understanding of biomechanical (e.g., spatiotemporal parameters, kinematics, kinetics) and neurophysiological adaptations (e.g., neuro-muscular activation, muscle-tendon unit behavior) related to BWS during different modes of locomotion (e.g., loping, skipping, running), movement (e.g., hopping, jumping) and % of body weight unloading;
- Improving our understanding of the association between BWS-induced reductions of external loading and changes in internal forces (e.g., forces and moments experienced at the joint and muscle);
- Improving our understanding of the interaction between supraspinal (e.g., corticomotor excitability) and spinal (e.g., CPGs) mechanisms during BWS locomotion.

## 5. Conclusion

The use of BWS devices is a valuable tool to increase our knowledge of biomechanical, physiological, and sensori-motor adaptations to partial body unloading. As a result, it has the potential to make important contributions to the optimization of spaceflight operations as well as terrestrial rehabilitation. Yet, current scientific contributions are heterogenous as protocols and reporting of data vary widely. Establishing guidelines for standardization of hypogravity-related research would greatly improve scientific advancements in both areas.

## Author contributions

ED, DG, DC, TW, and NH: conceptualization, revision, and editing. ED and NH: writing first draft. All authors have read and agreed to the published version of the manuscript. All authors contributed to the article and approved the submitted version.
